# Usefulness of expanding the indications of early rescue intracytoplasmic sperm injection

**DOI:** 10.1002/rmb2.12432

**Published:** 2021-12-13

**Authors:** Takashi Shibahara, Yuu Fukasaku, Nozomi Miyazaki, Hiroaki Kawato, Hiroyuki Minoura

**Affiliations:** ^1^ Minoura Ladies Clinic Suzuka Japan; ^2^ Kawato Ladies Clinic Yokkaichi Japan

**Keywords:** blastocyst, fertilization, intracytoplasmic sperm injection, oocyte, polar body

## Abstract

**Purpose:**

Early rescue intracytoplasmic sperm injection (ICSI) is often performed in cases in which not even a single oocyte has extruded a second polar body 6 h after insemination. We evaluated the usefulness of expanding the indications of early rescue ICSI to cases in which <80% of oocytes have extruded second polar bodies 6 h after insemination.

**Methods:**

Early rescue ICSI was performed on oocytes that were denuded 2.5 h post‐insemination and whose extrusion of the second polar bodies had been examined 6 h post‐insemination with a PolScope.

**Results:**

In vitro fertilization was performed on 24 496 oocytes of 4944 cycles, and 1438 cycles had <80% rate of the second polar body extrusion. Rescue ICSI was performed on 3933 oocytes. Three pronuclei (3PN) incidence of rescue ICSI was 3.0% in oocytes with ≥50% rate of the second polar body extrusion. With respect to the second polar body extrusion rate, no differences were observed in normal fertilization, blastocyst development, implantation, miscarriage, or live birth rates for rescue ICSI.

**Conclusion:**

By expanding the indications of early rescue ICSI using the PolScope to cases in which <80% of oocytes have extruded the second polar bodies, many fertilized oocytes can be obtained without considerably increasing the 3PN rate.

## Graphical abstract

Evaluation of the usefulness of expanding indications of eary rescue ICSI to case where <80% of oocytes have released second polar bodies 6 h after insemination. By expanding indivations of early resucue ICSI using the Polscope, many fertilized oocyte are obtained without greatly increasing the 3PN rate.

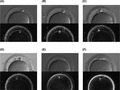

## 1 INTRODUCTION

In vitro fertilization (IVF) is the first‐choice fertilization method; however, it has drawbacks such as 5%–20% total fertilization failure (TFF)^1^ and lower fertilization rate than intracytoplasmic sperm injection (ICSI).^2^ For improving the fertilization rate, rescue ICSI is performed on oocytes that have not been fertilized in IVF. The earliest attempt was reported by Nagy et al. in 1993^3^ on rescue ICSI performed on the day after oocyte retrieval. Several follow‐up studies concluded that the pregnancy rate was low owing to oocyte aging.^4^, ^5^ In 2003, Chen et al. performed rescue ICSI on oocytes from which the second polar bodies had not been extruded 6 h after insemination, achieved a high pregnancy rate, and reported the usefulness of early rescue ICSI.^6^

Early rescue ICSI is indicated for cases suspected of TFF, in which not even a single oocyte from which a second polar body has been extruded 4–8 h after insemination is obtained.^6^, ^7^, ^8^, ^9^, ^10^, ^11^, ^12^ Although studies reported that rescue ICSI was performed in cases where the second polar bodies had been extruded from some oocytes,^13^, ^14^, ^15^, ^16^ they targeted cases in which the ratio of oocytes from which the second polar bodies had been extruded was <50%. It is expected that many unfertilized oocytes can be saved, but a study reported a high polyspermy rate in rescue ICSI performed in cases with an IVF fertilization rate of >25%,^14^ thus raising concerns that unnecessary rescue ICSI may be performed if the indications are expanded.

When performing rescue ICSI on the day after oocyte retrieval, determining whether to perform the procedure based on the presence or absence of pronuclei (PN) is relatively simple. Early rescue ICSI, however, requires the physician to make a decision based on the number of polar bodies. Determining the number of polar bodies for oocytes with fragmented polar bodies is difficult, and performing unnecessary rescue ICSI on oocytes from which the second polar bodies have been extruded is problematic because it results in the formation of 3PN. Therefore, rescue ICSI is often indicated for TFF. To overcome the difficulty in determining the number of polar bodies, the spindle is observed to assist in the determination of second polar body extrusion.^17^, ^18^ However, few studies have examined this aspect, and no study has examined a large number of cases.

The present study examined the usefulness of expanding the indications of rescue ICSI while suppressing the risk of polyspermy by observing the spindle using PolScope and accurately determining the extrusion of second polar bodies. As the expansion of the indications of rescue ICSI to all oocytes from which second polar bodies have not been extruded has a small effect considering the increased effort required, in order to obtain >80% of fertilized oocytes, equivalent to ICSI, the indications of rescue ICSI were expanded to cases in which <80% of oocytes had extruded second polar bodies.

## 2 MATERIALS AND METHODS

### 2.1 Patients

A retrospective cohort study was conducted on 4944 IVF cycles and 2302 ICSI cycles from which metaphase (M) II oocytes were collected following oocyte retrieval in 2010–2019. Written patient consent was obtained after providing a thorough explanation. This study was conducted with the approval of the ethics committee of Minoura Ladies Clinic (Approval number: H21.03).

### 2.2 Stimulation, oocyte retrieval, and insemination

Controlled ovarian stimulation was performed mainly by the agonist/human menopausal gonadotropin (hMG) method, antagonist/hMG method, and clomiphene citrate/antagonist/hMG method. Oocyte retrieval was performed under the guidance of transvaginal ultrasound 36–38 h after the trigger by hCG or GnRHa agonist.

While examining oocytes, cumulus‐oocyte complexes were stretched, and the presence or absence of the first polar body was confirmed using an inverted microscope (IX70, Olympus). The oocytes were divided into MI oocytes and MII oocytes. Among the MII oocytes, the oocytes with fragmented polar bodies that appeared to have divided into two parts were separated and cultured. After oocyte retrieval, a maximum of five oocytes were added to the Center Well Dish containing 1 ml of human tubal fluid medium (Irvine Scientific, Santa Ana, USA) and then cultured.

For IVF, insemination was performed at least 3 h after preculture following oocyte retrieval. Standard insemination sperm concentration was set at 200 000/ml and corrected based on normal sperm morphology ratio and previous fertilization rate. ICSI was selected for cases where the adjusted motile sperm count was insufficient for the sperm count for insemination, as well as for cases in which TFF had occurred in a previous round of IVF. ICSI was performed at least 3 h after preculture.

### 2.3 Denudation in IVF, judgment of second polar bodies, and implementation of rescue ICSI

The denudation of cumulus‐oocyte complexes began 2.5 h after insemination. The denuded oocytes were added to a microdrop medium prepared with 35 μl lof mHTF culture medium (Irvine Scientific, Santa Ana) in a glass bottom Petri dish (D11140H, Matsunami Glass). For the oocytes, the extrusion of second polar bodies and state of spindle was observed using an inverted microscope (IX70, Olympus) equipped with a PolScope (Oosight Imaging System, Hamilton Thorne).

**FIGURE 1:**
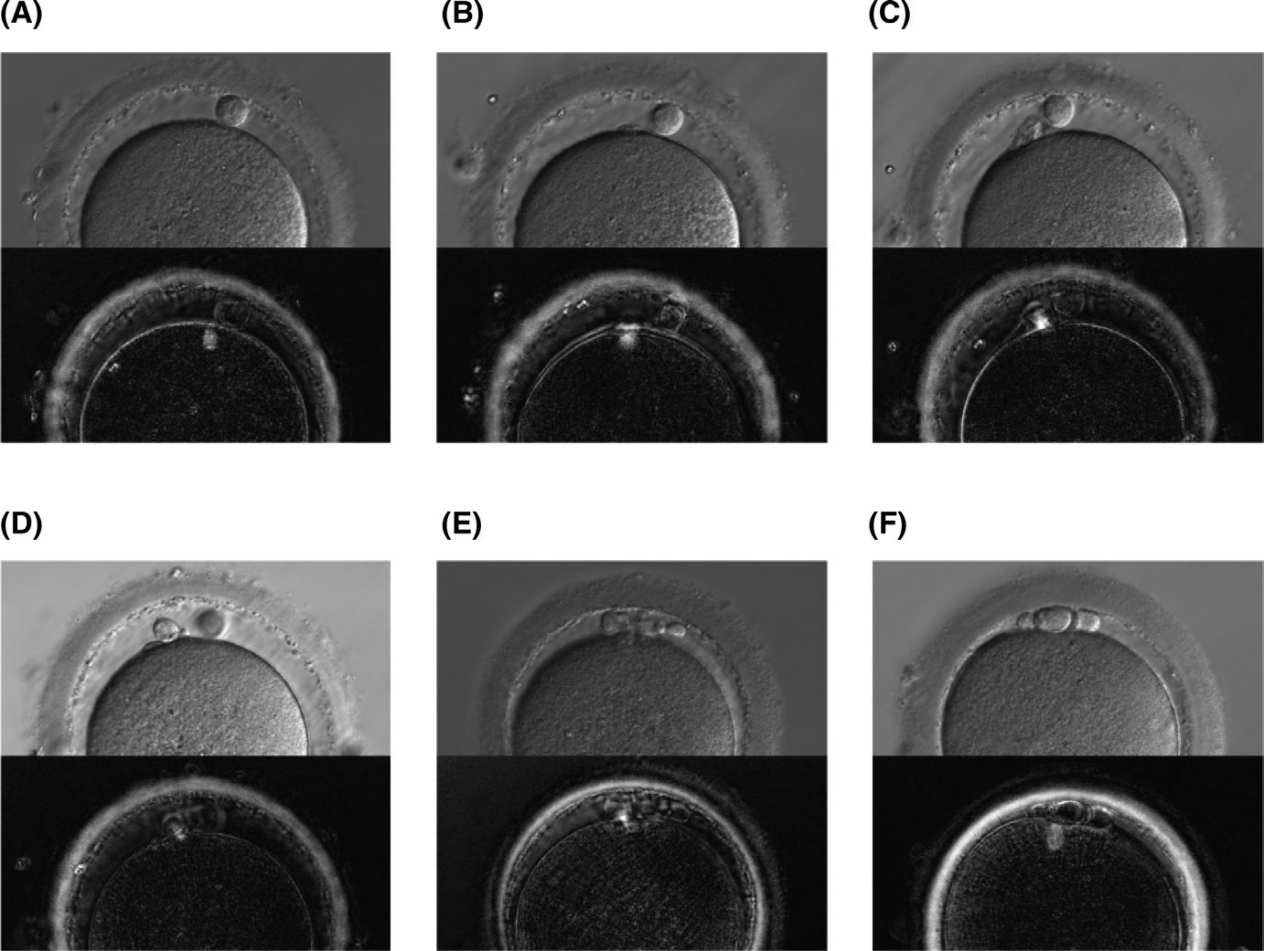
Polar bodies and spindles observed by relief contrast and PolScope. a, b, c, and d show changes over time until the second polar body is extruded, with the (A) spindle in the cytoplasm, (B) spindle in the protruded part on the cell membrane surface, (C) spindle cross‐linked between the second polar body and cytoplasm, and (D) spindle that is thinning and disappearing. (E) and (F) show the judgment of fertilization by spindle in cases where the number of polar body is difficult to determine, with (E) 2PB with cross‐linked spindle and (F) 1PB with spindle in the cytoplasm despite fragmented polar body

The spindle before and after the extrusion of second polar bodies is observed using the PolScope, and the spindle in the cytoplasm (Figure 1a) is observed at the protruded part on the cell membrane surface as fertilization progresses, and the second polar body begins to be extruded (Figure 1b). While the second polar body is being extruded, the spindle is observed to be cross‐linked between the second polar body and the cytoplasm (Figure 1c). After the second polar body is extruded, the spindle becomes thin (Figure 1d) and eventually disappears. For oocytes that extrude a fragmented first polar body and the number of polar bodies is difficult to determine, the extrusion of the second polar body can also be accurately judged by the state of the spindle (Figure 1e,f). The number of second polar bodies was determined including the state of the spindle.

In the assessment performed immediately after denudation, apart from oocytes with one polar body (1PB), an oocyte in which the spindle was observed in the cytoplasm despite the presence of a fragmented polar body, was determined as 1PB. An oocyte with two polar bodies (2PB), as well as a cross‐linked or disappearing spindle, was determined as 2PB. An oocyte without a spindle in which the first polar body was not fragmented during oocyte retrieval, clearly appearing to be two polar bodies, was determined as 2PB.

Oocytes in which polar bodies were observed were transferred to a microdrop medium prepared with 35 μl of global medium (Life Global, Brussels, Belgium) and then cultured in an incubator (APM‐30D, Astec, Fukuoka, Japan) at 37°C, 5% CO_2_, 5% O_2_, and 90% N_2_.

Oocytes determined to be 1PB immediately after denudation were observed again at 6 h after insemination to count the number of polar bodies. Oocytes where fragmentation occurred after denudation and in which the number of polar bodies was difficult to determine were compared with images obtained immediately after denudation, and oocytes in which the number of polar bodies clearly increased were determined as 2PB. When the number of polar bodies was ambiguous, the spindle was observed, and if the spindle remained, they were determined to be 1PB. However, if the spindle was cross‐linked or disappearing, they were determined as 2PB. For oocytes determined as 1PB, the spindle was confirmed again before performing rescue ICSI.

Indications of rescue ICSI were cycles with <80% of oocytes determined as 2PB 6 h after insemination. The extrusion rate of the second polar body was defined as the ratio of inseminated oocytes that were observed as having extruded the second polar body when determining whether or not to perform rescue ICSI. Even if the oocytes were determined as 1PB, oocytes that were 1PB during denudation but in which the spindle was not observed, those in which the spindle was in the central cytoplasm, and those with poor cytoplasmic conditions (such as atrophied cytoplasm and constricted cytoplasm) were considered poor quality oocytes and were excluded from the target of rescue ICSI in cases where at least 50% of oocytes had extruded second polar bodies.

### 2.4 Blastocyst culture and transfer

Fertilization was judged by the number of PN on the day after oocyte retrieval. A total of 99.1% (25634/25881) of 2PN embryos (excluding embryos that had undergone cleavage‐stage embryo transfer) were cultured into blastocysts. For blastocyst transfer, freeze‐thaw blastocyst transfer was performed in 98.7% (6320/6404) of cycles, except cycles where fresh blastocyst transfer had been performed, while single blastocyst transfer was performed in 96.5% (6101/6320). Blastocyst freezing was performed on embryos expanded to Gardner classification ≥2BB on the afternoon of Day 4 and ≥3 on Day 5 or 6.^19^ Thawed embryo transfer was performed 4–20 h after thawing in the ovulation cycle or hormone regulation cycle. Transferred embryos ≥4AA were graded as good, ≥3BB as fair, and <3BB as poor. Embryos transferred on the sixth day from oocyte retrieval were graded as good for ≥5AA, fair for ≥4BB, and poor for <4BB. Evaluation for implantation rates only included single blastocyst transfers.

### 2.5 Statistical analysis

The Kruskal‐Wallis test and the Bonferroni method for multiple comparison were performed for age. Logistic regression analysis was performed for fertilization and blastocyst development rates with oocyte age added as the explanatory variable, and logistic regression analysis was performed for implantation rates, miscarriage rates, and live birth rates, with oocyte age and transferred blastocyst grading added as the explanatory variables. A *p*‐value <0.05 was determined to be statistically significant. Software R version 3.4.1 was used for statistical analysis.

## 3 RESULTS

**FIGURE 2:**
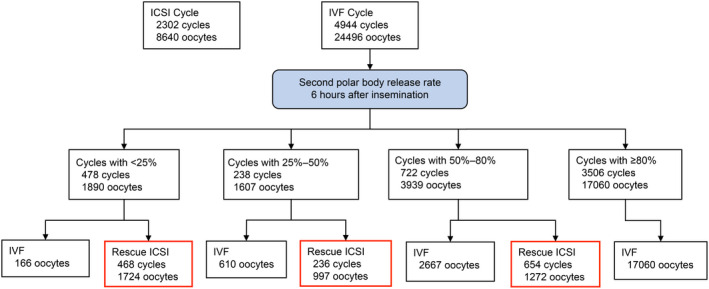
Classification of oocytes by the second polar body extrusion rate for IVF cycles

ICSI was performed on 8640 oocytes of 2302 cycles, while IVF was performed on 24496 oocytes of 4944 cycles. Rescue ICSI was performed on 3993 (16.3%) oocytes of 1358 (27.5%) cycles. Regarding the second polar body extrusion rate, the following findings/results were observed: 478 (9.7%) cycles had a second polar body extrusion rate of <25%, and rescue ICSI was performed on 1724 of 1890 oocytes; 238 (4.8%) cycles had a second polar body extrusion rate of 25%–50%, and rescue ICSI was performed on 997 of 1607 oocytes; 722 (14.6%) cycles had a second polar body extrusion rate of 50%–80%, and rescue ICSI was performed on 1272 of 3939 oocytes; and 3506 cycles had a second polar body extrusion rate of ≥80% with 17060 oocytes, and rescue ICSI was not performed (Figure 2).

Among 24496 oocytes that underwent IVF, 11865 oocytes (48.4%) were determined to be 2PB immediately after denudation performed 2.5–3.5 h after insemination. In terms of breakdown of the spindle state at that time, the spindle was cross‐linked between the second polar body and cytoplasm in 11553 oocytes (47.2%), thin and disappearing in 265 oocytes (1.1%), and not observed in 47 oocytes (0.2%). Moreover, 12505 oocytes (51.0%) were determined as 1PB, the spindle was in the cytoplasm in 10673 oocytes (43.6%), the spindle was in the protruded part on the cell membrane surface in 1261 oocytes (5.1%), and 571 oocytes (2.3%) had no spindle.

**FIGURE 3:**
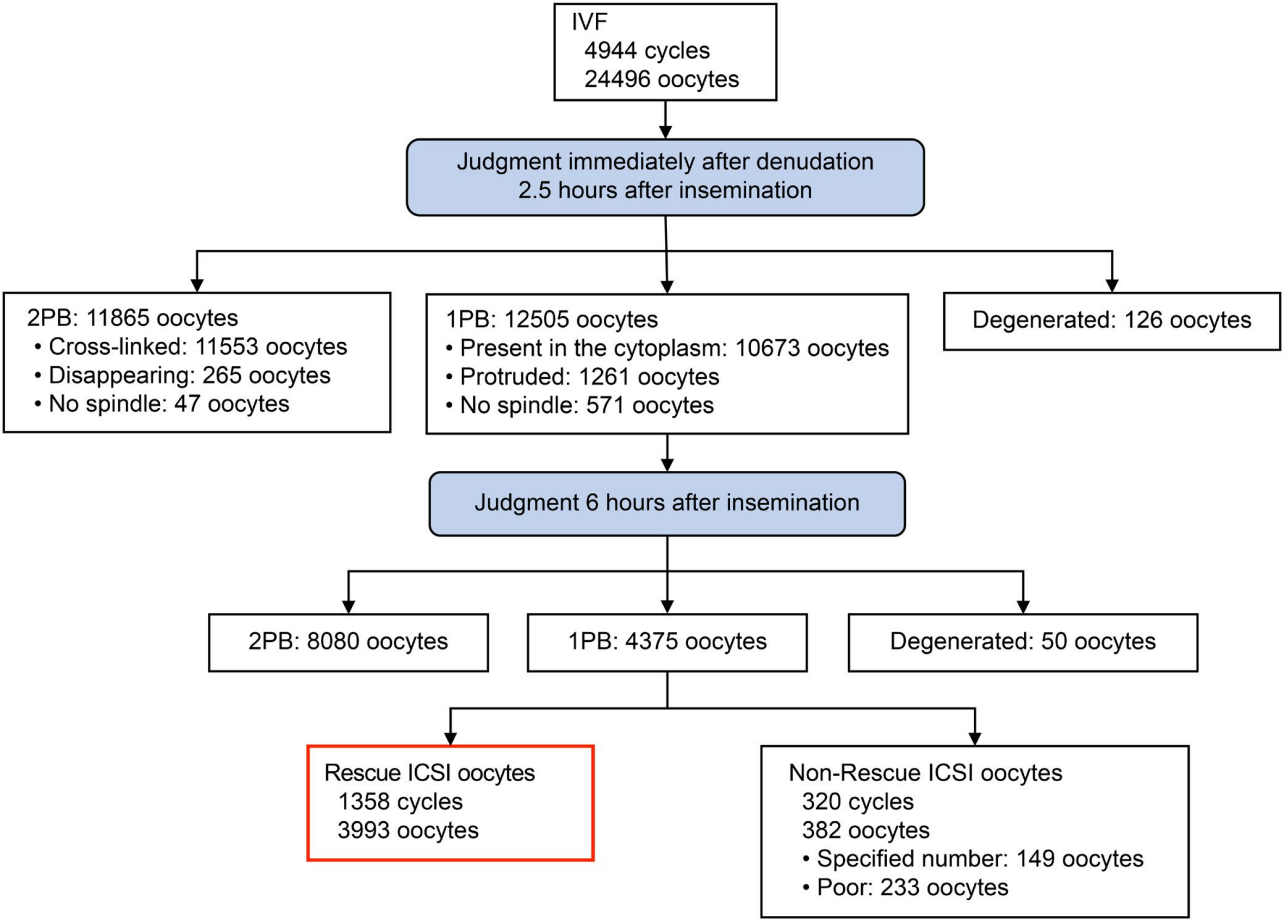
Classification of polar bodies in IVF cycles and the implementation of rescue ICSI. “Present in the Cytoplasm” corresponds to Figure 1A, “Protruded” corresponds to Figure 1B, “Cross‐linked” corresponds to Figure 1C, and “Disappearing” corresponds to Figure 1D

Among oocytes determined as 1PB immediately after denudation, the second polar body were extruded 6 h after insemination from 8080 oocytes (33.0%), and 4375 oocytes (17.9%) were ultimately determined as 1PB. Among oocytes determined as 1PB, rescue ICSI was performed on 3993 oocytes (16.3%) of 1358 cycles (27.5%). Rescue ICSI was not performed on 382 oocytes (1.6%) of 320 cycles (6.5%), even though they had been judged as 1PB. Among them, 149 (0.6%) had a second polar body extrusion rate of ≥80%, and 233 (1.0%) had a poor oocyte quality; thus, rescue ICSI was not performed (Figure 3).

Table 1 shows the comparison by fertilization methods. Compared with that of rescue ICSI (76.3%), the 2PN rate was higher in IVF (79.3%) (*p* < 0.001) and showed no difference from that of ICSI. Compared with that of rescue ICSI (2.2%), the 3PN rate was higher in IVF (9.8%) (*p* < 0.001) and lower in ICSI (1.6%) (*p* < 0.011). Compared with that of rescue ICSI (11.4%), the 0PN rate was lower in IVF (2.9%) (*p* < 0.001) and showed no difference from that of ICSI (11.9%). Compared with rescue ICSI (50.8%), the Day 5 blastocyst development rate was higher for IVF (60.6%) and ICSI (53.5%) (*p* < 0.001). Compared with rescue ICSI (57.3%), Day 6 blastocyst development rate was higher in IVF (69.7%) (*p* < 0.001) but showed no difference from ICSI (58.6%). Compared to rescue ICSI, the grade of transferred embryos was good in IVF (*p* < 0.001), but when analysis was conducted by inputting grade as the explanatory variable, there were no differences in the implantation, miscarriage, or live birth rates.

**TABLE 1:** Comparison of ICSI, IVF, and rescue ICSI oocytes using the fertilization method

	ICSI oocytes	IVF oocytes	Rescue ICSI oocytes	*p*‐value Rescue ICSI vs. ICSI	*p*‐value Rescue ICSI vs. IVF
No. of cycles	2302	3506	1358		
No. of oocytes	8640	17060	3993		
Oocyte age	36.0 ± 4.7	36.0 ± 4.1	35.8 ± 4.2	<0.001	<0.11
Total motile sperm count (million/ml)	12.1 ± 20.0	46.2 ± 34.0	38.3 ± 30.0	<0.001	<0.001
1PN (%)	362 (4.2%)	650 (3.8%)	170 (4.3%)	0.86	0.14
2PN (%)	6546 (75.8%)	13527 (79.3%)	3045 (76.3%)	0.60	<0.001
3PN (%)	140 (1.6%)	1672 (9.8%)	87 (2.2%)	0.025	<0.001
0PN (%)	1027 (11.9%)	499 (2.9%)	454 (11.4%)	0.40	<0.001
No. of blastocyst cultures	6454	13496	3022		
Day 5 blastocysts (% per 2PN)	3433 (53.3%)	8183 (60.6%)	1534 (50.8%)	0.014	<0.001
Day 6 blastocysts (% per 2PN)	3779 (58.6%)	9406 (69.7%)	1733 (57.3%)	0.23	<0.001
No. of single blastocyst transfers	1387	3418	590		
Patient age during oocyte retrieval	35.3 ± 3.9	35.6 ± 4.1	35.4 ± 4.3	1.00	1.00
Transferred blastocyst grading				0.55	<0.001
Good	452 (32.6%)	1548 (45.3%)	160 (27.1%)		
Fair	607 (43.8%)	1360 (39.8%)	298 (50.5%)		
Poor	328 (23.6%)	510 (14.9%)	132 (22.4%)		
Implantation (% per BT)	644 (46.9%)	1691 (49.5%)	252 (43.2%)	0.28	0.25
Miscarriage (% per implantation)	131 (20.3%)	381 (22.5%)	45 (17.6%)	0.35	0.13
Live births (% per BT)	510 (36.8%)	1293 (37.8%)	204 (34.6%)	0.65	0.98

Abbreviations: BT, blastocyst transfer; ICSI, intracytoplasmic sperm injection; IVF, in vitro fertilization; PN, pronuclei.

Table 2 shows the comparison by second polar body extrusion rate of rescue ICSI oocytes. 3PN rate showed no difference in cycles with a second polar body extrusion rate of <25% and 25%–50% but was significantly higher in that of ≥50% (*p* = 0.025). There was no difference in the blastocyst development rate, implantation rate, miscarriage rate, and live birth rate.

**TABLE 2:** Comparison of rescue ICSI oocytes by second polar body extrusion rate

	Cycles with <25%	Cycles with 25%–50%	Cycles with 50%–80%	*p*‐value
No. of rescue ICSI cycles	468	236	654	
No. of rescue ICSI oocytes	1724	997	1272	
Oocyte age	35.9 ± 4.3	35.6 ± 4.2	35.9 ± 4.1	0.37
1PN (%)	73 (4.2%)	48 (4.8%)	49 (3.9%)	0.36
2PN (%)	1340 (77.6%)	752 (75.4%)	953 (74.9%)	0.15
3PN (%)	31(1.8%)	18 (1.8%)	38 (3.0%)	0.025
0PN (%)	202 (11.7%)	119 (11.9%)	133 (10.5%)	0.25
No. of blastocyst cultures	1,319	752	951	
Day 5 blastocysts (% per 2PN)	692 (52.4%)	383 (50.9%)	459 (48.3%)	0.11
Day 6 blastocysts (% per 2PN)	782 (59.3%)	424 (56.4%)	527 (55.4%)	0.10
No. of single blastocyst transfers	300	131	159	
Patient age during oocyte retrieval	35.6 ± 4.3	35.0 ± 4.3	35.6 ± 4.2	0.67
Transferred blastocyst grading				0.42
Good	82 (27.3%)	39 (29.8%)	39 (24.5%)	
Fair	143 (47.7%)	61 (46.6%)	94 (59.2%)	
Poor	75 (25.0%)	31 (23.7%)	26 (16.4%)	
Implantation (% per BT)	123 (40.3%)	65 (49.6%)	67 (42.1%)	0.32
Miscarriage (% per implantation)	23 (19.0%)	12 (18.5%)	10 (14.9%)	0.53
Live births (% per BT)	97 (32.3%)	51 (38.9%)	56 (35.2%)	0.24

Abbreviations: BT, blastocyst transfer; ICSI, intracytoplasmic sperm injection; PN, pronuclei.

Table 3 shows the comparison of non‐rescue ICSI oocytes, which did not undergo rescue ICSI despite being judged as 1PB, after they had been divided into good oocytes that did not undergo rescue ICSI as the second polar body extrusion rate had reached the specified number of ≥80% 6 h after insemination, and poor oocytes that did not undergo rescue ICSI owing to abnormal spindle or poor cytoplasm quality. The 2PN rate of good oocytes was 4.0% and that of poor oocytes was 10.8%. Compared with good oocytes, the abnormal fertilization rates of 1PN (*p* = 0.019) and 3PN (*p* < 0.001), as well as 0PN (*p* < 0.001), were higher in poor oocytes.

**TABLE 3:** Comparison of non‐rescue ICSI oocytes by embryo quality

	Good oocytes	Poor oocytes	*p*‐value
No. of cycles	130	195	
No. of oocytes	149	233	
Oocyte age	35.3 ± 4.3	36.3 ± 3.7	0.021
1PN (%)	1 (0.67%)	20 (8.6%)	0.019
2PN (%)	6 (4.0%)	21 (10.8%)	0.065
3PN (%)	1 (0.67%)	61 (26.2%)	<0.001
0PN (%)	137 (91.9%)	113 (48.5%)	<0.001
No. of blastocyst cultures	6	21	
Day 6 blastocysts (% per 2PN)	2 (33.3%)	3 (14.2%)	0.47

Abbreviation: PN, pronuclei.

## 4 DISCUSSION

The indications of rescue ICSI were expanded to cases with <80% of oocytes in the cycle having extruded the second polar body 6 h after insemination, but there was no difference in 2PN, blastocyst development, implantation, miscarriage, and live birth rates even in cases with a second polar body extrusion rate of 50% or more. According to Cao et al., the 3PN rate of rescue ICSI increased as the IVF fertilization rate increased, with 8.6% for an IVF fertilization rate of 0%, 11.3% for that of <25%, and 14.5% for that of 25%–50%.^14^ In this study, there was no difference in the 3PN rate between a second polar body extrusion rate of <25% and 25%–50%, whereas the 3PN rate increased for that of ≥50% but remained low at 3.0%. Moreover, the 0PN rate for oocytes that did not undergo rescue ICSI despite being 1PB as the second polar body extrusion rate had reached the specified number of ≥80% was 91.9%; many oocytes determined as 1PB were not fertilized. Oocytes that underwent rescue ICSI with a second polar body extrusion rate of 25%–50% accounted for 4.1% of oocytes that underwent IVF, whereas oocytes with a second polar body extrusion rate of 50%–80% accounted for 5.2%. The expansion of rescue ICSI indications to <50% can rescue many oocytes without increasing the 3PN rate, and the expansion to <80% can also rescue many oocytes without increasing the 3PN rate significantly.

An issue with the fertilization of rescue ICSI is the difficulty in determining the number of polar bodies. The implementation of rescue ICSI following the misjudgment of 1PB as 2PB, which has extruded the second polar body, forms 3PN. The misjudgment of the fragmented 1PB as 2PB, even when the second polar body has not been extruded, results in failed fertilization. According to Nagy et al., 18.2% of oocytes with fragmented polar bodies that did not undergo rescue ICSI were fertilized,^20^ which exemplifies how difficult it is to accurately determine the number of polar bodies for oocytes with fragmented polar bodies. The 3PN rate of early rescue ICSI has been reported to be 1.6%–9.4%.^6^, ^7^, ^8^, ^17^ The 3PN rate of rescue ICSI in this study was 2.2%, which is higher than 1.6% in ICSI but still low. The low 3PN rate is attributed in part to the accurate assessment of second polar body extrusion in oocytes with fragmented polar bodies by additionally assessing the spindle state. Guo et al. have reported the usefulness of observing the spindle in early rescue ICSI and that the 3PN rates were 5.9% when the spindle was observed, and 9.4% when it was not observed.^17^ Moon et al. have also reported the usefulness, with a 3PN rate of 4.5% when the spindle is observed and 26.5% when the spindle is not observed on the day after insemination.^21^ The second reason for the low 3PN rate was polar body observation and MI/MII oocyte classification during oocyte retrieval. It has been reported that the fertilization rate decreases for a preculture time <3 h,^22^, ^23^ and normal fertilization may not progress despite sperm penetration in MI oocytes or oocytes that have just matured into MII. In standard IVF, insemination is performed 3 h after preculture without observing polar body during oocyte retrieval, but some oocytes in MI during oocyte retrieval do not mature into MII before insemination and mature into MII before denudation. Some of those oocytes do not undergo normal fertilization and do not extrude second polar body even if penetrated by the sperm and may become 3PN if rescue ICSI is performed. In this study, the observation of polar body during oocyte retrieval and exclusion of MI oocytes from the target of rescue ICSI may also have contributed to lower rates of 3PN.

However, the 3PN rate of rescue ICSI was higher than that of ICSI and even higher in cases with a high second polar body extrusion rate. If rescue ICSI is performed on oocytes with a late extrusion of second polar body even if penetrated by the sperm, 3PN may be formed. Among good oocytes that did not undergo rescue ICSI despite being 1PB as the ratio of oocytes having extruded second polar body had reached the specified number, 4.0% of the oocytes were fertilized normally the next day. There are also a few oocytes that are late extruders, extruding the second polar body later than 6 h after insemination; thus, it is considered difficult to completely eliminate unnecessary rescue ICSI. In addition, poor quality oocytes with poor cytoplasmic conditions are fertilized to a certain degree without rescue ICSI, but it is better to exclude them from the target of rescue ICSI because of the large number of abnormally fertilized oocytes.

In rescue ICSI, the decrease in embryo development owing to oocyte aging associated with the delayed timing of fertilization has been a concern. The fertilization and pregnancy rates have reportedly reduced when the time from oocyte retrieval to ICSI was prolonged,^24^, ^25^, ^26^ but it has also been reported that no effects were observed up to 9 h^27^ and 12 h^28^; as such, there is no consensus on the impact of delayed timing of ICSI implementation on embryo development. Studies on embryo development in early rescue ICSI have reported grades of cleavage stage embryos lower than IVF^20^ or equal to ICSI,^8^, ^17^ but there are few studies on their development into blastocysts. In this study, Day 5 blastocyst development rate was lower than that of ICSI oocytes, but Day 6 blastocyst development rate showed no difference. In rescue ICSI, Day 5 blastocyst development rate is low owing to delayed fertilization timing, but there is no difference in the final Day 6 development rate, showing the same embryo development as in ICSI, suggesting that the aging of oocytes may not have a great impact. However, it has been reported that compared with rescue ICSI, sperm findings are poor in ICSI, and embryo development decreases with the decrease in semen findings ^29^, ^30^, so embryo development in rescue ICSI may in fact not be completely equal to ICSI. In terms of embryo development after rescue ICSI performed based on the second polar body extrusion rate, Cao et al. reported that the higher the second polar body extrusion rate, the lower the blastocyst development rate.^14^ In our study, however, the blastocyst development and implantation rates did not decrease in cycles with a second polar body extrusion rate of ≥50%, indicating that the expansion of rescue IVF indications does not exert a negative effect on embryo development.

For early rescue ICSI that checks the first polar body at the time of oocyte retrieval and observes the spindle immediately after denudation and 6 h after, the expansion of indications to cases with a second polar body extrusion rate of <80% can enable the efficient rescue of unfertilized oocytes without significantly increasing the 3PN rate. When taking into account embryo development and live birth rates, it appears useful to expand the indications of rescue IVF. However, for cases with a high second polar body extrusion rate, the decision to perform rescue ICSI needs to be made individually for each case and institution taking into account the slightly increased possibility of 3PN.

## CONFLICTS OF INTEREST

Takashi Shibahara, Yuu Fukasaku, Nozomi Miyazaki, Hiroaki Kawato, and Hiroyuki Minoura declare that they have no conflict of interest.

## HUMAN RIGHTS STATEMENTS AND INFORMED CONSENT

All procedures followed were in accordance with the ethical standards of the responsible committee on human experimentation (institutional and national) and with the Helsinki declaration of 1964 and its later amendments. Informed consent was obtained from all patients for being included in the study.

## ETHICS APPROVAL

Approval was obtained from the ethics committee of Minoura Ladies Clinic (Approval number: H21.03). The procedures used in this study adhere to the tenets of the declaration of Helsinki.

## INFORMED CONSENT

Written patient consent was obtained after providing a thorough explanation.
